# Dietary Inflammatory Index and Cardiovascular Risk and Mortality—A Meta-Analysis

**DOI:** 10.3390/nu10020200

**Published:** 2018-02-12

**Authors:** Nitin Shivappa, Justyna Godos, James R. Hébert, Michael D. Wirth, Gabriele Piuri, Attilio F. Speciani, Giuseppe Grosso

**Affiliations:** 1 Cancer Prevention and Control Program, University of South Carolina, Columbia, SC 29208, USA; JHEBERT@mailbox.sc.edu (J.R.H.); wirthm@mailbox.sc.edu (M.D.W.); 2 Department of Epidemiology and Biostatistics, Arnold School of Public Health, University of South Carolina, Columbia, SC 29208, USA; 3 Connecting Health Innovations, LLC, Columbia, SC 29201, USA; 4 NNEdPro Global Centre for Nutrition and Health, St John’s Innovation Centre, Cambridge CB4 0WS, UK; justynagodos@gmail.com (J.G.); giuseppe.grosso@studium.unict.it (G.G.); 5 College of Nursing, University of South Carolina, Columbia, SC 29208, USA; 6 Inflammation Society, 18 Woodlands Park, Bexley DA52EL, UK; gabriele.piuri@studiospeciani.it (G.P.); attilio.speciani@me.com (A.F.S.); 7 Integrated Cancer Registry of Catania-Messina-Siracusa-Enna, Azienda Ospedaliera Universitaria Policlinico Vittorio Emanuele, 95123 Catania, Italy

**Keywords:** diet, cytokines, nutrition, inflammation, epidemiology, dietary inflammatory index, cardiovascular diseases, meta-analysis

## Abstract

Diet and chronic inflammation have been suggested to be risk factors in the development of cardiovascular disease (CVD) and related mortality. The possible link between the inflammatory potential of diet measured through the Dietary Inflammatory Index (DII^®^) and CVD has been investigated in several populations across the world. The aim of this study was to conduct a meta-analysis on studies exploring this association. Data from 14 studies were eligible, of which two were case-control, eleven were cohort, and one was cross-sectional. Results from the random-effects meta-analysis showed a positive association between increasing DII, indicating a pro-inflammatory diet, and CVD. Individuals in the highest versus the lowest (reference) DII category showed a 36% increased risk of CVD incidence and mortality, with moderate evidence of heterogeneity (relative risk (RR) = 1.36, 95% confidence interval (CI): 1.19, 1.57; heterogeneity index *I*^2^ = 69%, *p* < 0.001). When analyzed as a continuous variable, results showed an increased risk of CVD risk and mortality of 8% for each one-point increase in the DII score. Results remained unchanged when analyses were restricted to the prospective studies. Results of our meta-analysis support the importance of adopting a healthier anti-inflammatory diet for preventing CVD incidence and related mortality. In conclusion, a pro-inflammatory diet is associated with increased risk of CVD and CVD mortality. These results further substantiate the utility of DII as tool to characterize the inflammatory potential of diet and to predict CVD incidence and mortality.

## 1. Introduction

Chronic inflammation is characterized by the continuous presence of pro-inflammatory cytokines through increased blood flow during tissue injury, as a consequence of histamine released by damaged mast cells [1], and is known to play a major role in the development of cardiovascular diseases (CVD) and related mortality [2]. Previous research has indicated that whole diet and various dietary components have a direct association with inflammation [3,4,5,6]. Consumption of fruit and vegetables has been shown to reduce levels of inflammation, while consumption of red meat has been shown to increase inflammation [7,8]. Various dietary components also have been implicated in playing a major role in the development of various CVDs [8,9,10]. In a recent meta-analysis, increased intake of processed meat has been shown to be associated with increased risk of CVD mortality [11]. Increased adherence to healthier dietary patterns characterized by increased intake of plant-based foods such as fruit and vegetables, nuts, and whole grains and adherence to a healthier dietary pattern has been shown to help prevent and to manage CVD [12,13,14]. Finally, an increased intake of polyphenols, anti-oxidants with ability to decreases oxidative stress and inflammation through scavenging free radicals [15], was shown to be associated with decreased risk of overall and CVD-related mortality [16].

Until 2009, there was no tool that could take into account the entire diet and determine its inflammatory potential. Researchers from University of South Carolina have developed a dietary tool called the Dietary Inflammatory Index (DII^®^) that places individuals on a continuum from maximally pro-inflammatory to maximally anti-inflammatory diet. The first version of DII was developed based on literature published on various dietary components and inflammation through 2007 [17]. In the first version, 927 peer reviewed articles were scored based on the direction and significance of the association between various dietary components and each of the six inflammatory markers (C-reactive protein (CRP); interleukin (IL)-6, -1β, -4, and -10; and tumor necrosis factor (TNF)-α). In 2014, the DII^®^ was refined and improved with literature review extending through 2010 [18], wherein the number of articles that were reviewed and scored increased to 1943. The DII categorizes individuals’ diets according to their inflammatory potential on a continuum from maximal pro-inflammatory to maximal anti-inflammatory. A higher DII score indicates a more pro-inflammatory diet, whereas a lower DII score represents a more anti-inflammatory diet. The DII can be calculated from various dietary assessment tools, with food frequency questionnaires (FFQs) being the most commonly used tool [19,20,21]. The DII has been construct validated in several studies, in the first validations study DII has been shown to be associated with changes in high-sensitivity-CRP in the Seasonal Variation in Blood Cholesterol Study [17,20]. Subsequently, the DII has been validated in several studies from around the world with different measures of inflammation such as CRP, IL-6, and TNF-α-R2 [19,22,23,24,25]. Apart from the validation studies, DII has also been shown to be associated with various chronic inflammation-related health outcomes such as cancer incidence [26,27,28], all-cause and cancer-specific mortality [29,30,31], respiratory conditions such as asthma [32,33] and cognitive disorders [34,35]. In a recent meta-analysis from nine studies that looked at the association between DII and colorectal cancer (CRC), individuals in the highest versus the lowest (reference) DII category showed an overall 40% increased risk of colorectal cancer (CRC), with moderate evidence of heterogeneity (relative risk (RR) = 1.40, 95% confidence interval (CI): 1.26, 1.55; *I*^2^ = 69%, *p* < 0.001) [36]. Several studies have found positive associations between DII score and CVD incidence and CVD mortality [9,10,14,37,38,39,40,41,42,43,44]. Using results from qualifying studies, the purpose of the current meta-analysis was to investigate the cumulative association between the inflammatory potential of diet, as measured by the DII score, and CVD risk and CVD mortality.

## 2. Methods

We followed the Meta-Analysis of Observational Studies in Epidemiology (MOOSE) protocol throughout the design, execution, analysis and reporting of the present meta-analysis [45].

### 2.1. Search Strategy and Study Selection

A comprehensive literature search was conducted using PubMed (National Library of Medicine) and Excerpta Medica database (EMBASE); databases were screened from the earliest available online indexing year up to September 2017, with Title/Abstract and MESH terms restriction. The following search terms were included: (dietary inflammatory index or inflammatory diet or anti-inflammatory diet or dietary score) and (CVD or cardiovascular disease or coronary heart disease (CHD) or ischemic heart disease (IHD) or myocardial infarction or stroke or heart attack or hypertension). Two authors performed the search independently. We included observational studies with any type of design (prospective, case-control and cross-sectional) that evaluated the association between the DII and cardiovascular disease risk and mortality. Studies were eligible if they provided corresponding risk estimates, such as RRs (risk ratios), HRs (hazard ratios), or ORs (odds ratios). We excluded studies that reported insufficient statistics or evaluated other dietary scores as exposure. Reference lists of included manuscripts also were “hand searched” for additional studies not previously identified. When a duplicate report from the same study/cohort was identified, we included the report that provided the largest number of cases/entire cohort or had the longest follow-up for each endpoint of interest. Two authors assessed full texts of potentially relevant articles independently.

### 2.2. Data Extraction

Data were extracted from each eligible study using a standardized extraction form. The following information was collected: (i) name of the first author; (ii) year of publication; (iii) study cohort or name; (iv) country; (v) number of participants; (vi) sex of participants; (vii) age range or mean age of the study population at baseline; (viii) follow-up period; (ix) endpoints and cases; (x) measures of risk or association with 95% confidence intervals (CIs) for the highest versus the lowest category of exposure and for one-point increase of the DII score (when available); and (xi) covariates used for adjustment.

The quality of eligible studies was assessed in accordance to the Newcastle–Ottawa Quality Assessment Scale [46], which consist of three parameters of quality: selection (four points), comparability (two points), and outcome (three points), with a score of seven or more points reflecting high quality.

### 2.3. Statistical Analysis

In this meta-analysis, ORs and HRs were deemed equivalent to RRs [47]. Random-effects models were used to calculate pooled RRs (with 95% CIs) of CVD incidence and CVD mortality for the highest compared to the lowest category of exposure and for a one-point increase in the DII score. Risk estimates of CVD occurrence and mortality for one-point increase of the DII score (continuous), were estimated for the studies not reporting the measure but providing sufficient data to estimate it. Heterogeneity was assessed using the *Q* test and *I*^2^ statistic. The significance for the *Q* test was defined as *p* < 0.10. The *I*^2^ statistic represents the amount of total variation that could be attributed to heterogeneity. *I*^2^ values ≤ 25%, 25–50%, 50–75% and >75% indicated no, small, moderate, and high heterogeneity, respectively. A sensitivity analysis was conducted by excluding one study at the time in order to assess the stability of results. Subgroup analyses were conducted by type of cardiovascular event, sex, geographical, and adjustment for body mass index (BMI), smoking, education and physical activity. Publication bias was assessed by visual observation of funnel plot. All analyses were performed with Review Manager (RevMan) version 5.2 (The Nordic Cochrane Centre, The Cochrane Collaboration, Copenhagen, Denmark).

## 3. Results

### 3.1. Study Characteristics

**Figure 1:**
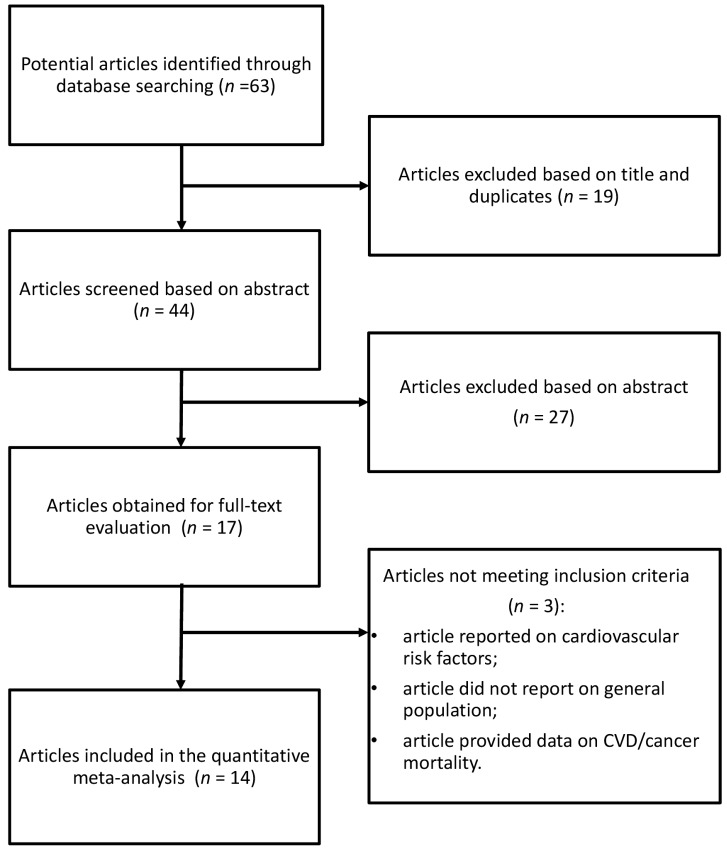
Selection of relevant studies reporting on the association between Dietary Inflammatory Index (DII^®^) and cardiovascular disease (CVD) occurrence and mortality.

The database search identified 63 studies, of which 19 were excluded after reviewing the title, and 27 based on abstract (Figure 1). Of the remaining 17 publications, three were excluded for the following reasons: (i) article reported on cardiovascular risk factors, not CVD events; (ii) article did not report on general population, it used a clinic population with possible prior CVD; and (iii) article provided data on total/all-cause mortality, from which CVD deaths could not be distinguished.

For the analysis on the association between DII and CVD risk and mortality, 14 studies were eligible [9,10,29,37,38,39,40,41,42,43,44,48,49,50]. Eleven reports were prospective studies [10,29,37,38,39,40,41,42,43,48,49], one study had cross-sectional design [50] and two studies were case-control [9,44]. Case-control studies were used only for the subgroup analysis of risk of myocardial infarction. Prospective studies included the following cohorts: the Iowa Women’s Health Study (IWHS), National Health and Nutrition Examination Survey (NHANES)-III study, Swedish Mammography Study, Whitehall II study, MONICA/KORA Augsburg study, Supplémentation en Vitamines et Minéraux AntioXydants (SU.VI.MAX), The Australian Longitudinal Study on Women's Health (ALSWH), Geelong Osteoporosis Study (GOS), Prevención con Dieta Mediterránea (PREDIMED), Seguimiento Universidad de Navarra (SUN) and Calcium Intake Fracture Outcome Study (CAIFOS). Studies eligible for the meta-analysis comprised 161,337 participants and 15,738 cases. Main characteristics of the studies included in the meta-analysis are described in Table 1. Six studies provided relative risk for CVD incidence [10,38,39,48,49,50], six for CVD mortality [29,37,40,41,42,43], three for CHD mortality [29,37,42], three for IHD/CHD [42,49,50], three for stroke [48,49,50], four for myocardial infarction [9,44,48,49], and two for angina pectoris [48,50]. Three studies were conducted in North America [29,43,50], eight in Europe [9,10,39,40,41,42,44,48] and three in Australia [37,38,49]. The follow-up time in prospective cohort studies ranged from about 4.3 to 26 years.

### 3.2. DII and CVD Risk and Mortality

**Figure 2:**
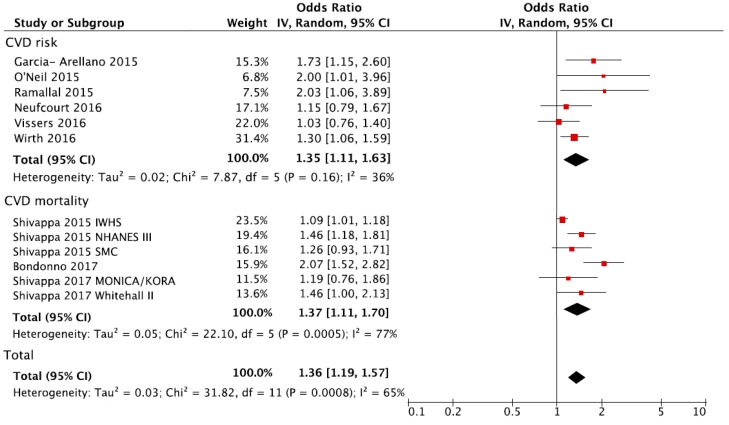
Forest plot of summary relative risks (RRs) of CVD occurrence and CVD mortality for the highest vs. lowest (reference) category of DII. IWHS: the Iowa Women’s Health Study; NHANES: National Health and Nutrition Examination Survey; SMC: Swedish Mammography Study.

**Figure 3:**
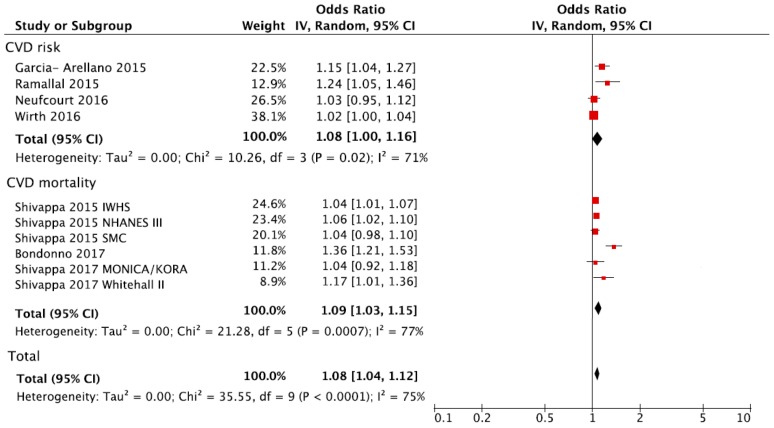
Forest plot of summary relative risks (RRs) of CVD occurrence and CVD mortality for one-point increase of DII.

The overall analysis of the association between the DII and CVD (either risk or mortality) is shown in Figure 2. Individuals in the highest versus the lowest (reference) DII group of exposure had a 36% increased risk of CVD (RR = 1.36, 95% CI: 1.19, 1.57). There was evidence of heterogeneity (*I*^2^ = 65%, *p* < 0.001) but no evidence of publication bias after examining the funnel plot (Figure S1). A sensitivity analysis conducted by excluding one study at a time revealed that the results from the IWHS were the largest contributor to the heterogeneity and its exclusion reduced heterogeneity to 38% (*p* = 0.10) with substantially unchanged effect size (RR = 1.41, 95% CI: 1.24, 1.61). The analysis considering the DII score as a continuous variable showed an increased risk of CVD of 8% for each one-point increase of the score (RR = 1.08, 95% CI: 1.04, 1.12) (Figure 3).

Despite that results were affected by heterogeneity, no publication bias was evident after examination of the funnel plot (Figure S2).

The analysis for CVD risk and CVD mortality showed similar effect sizes (Figure 2), with a substantial increased risk of events in individuals with the highest DII compared to the lowest group (Table 2). However, results for CVD mortality were affected by high heterogeneity (*I*^2^ = 77%, *p* < 0.001). Assessment of risk estimates for individual outcomes among CVD showed no significant results besides the association between DII and myocardial infarction; however, statistical power of these analyses was limited due to the reduced number of available datasets (Table 2).

### 3.3. Subgroup Analyses

Several subgroups were conducted to assess whether some variables of interest might be responsible for the heterogeneity observed between results of studies exploring the relation between DII and CVD (either risk or mortality; Table 3). The analysis revealed that increased risk of CVD in individuals with high versus low DII was significant only in women and in studies conducted in Europe and North America, but not in men and studies conducted in Australia (Table 3). Moreover, among the variables investigated, adjustment for BMI and physical activity was found to be crucial for obtaining significant results compared to studies that did not adjust for such variables (Table 3).

## 4. Discussion

This meta-analysis, which used results from 14 studies that have examined the association between the DII and CVD, showed strong evidence of association between increasing inflammatory potential of diet and CVD risk and related mortality. Therefore, increasing intake of healthy and anti-inflammatory dietary components such as fruits and green leafy vegetables and decreasing intake of pro-inflammatory components such as processed meat and sugar-sweetened drinks may play a vital role in reducing the risk of CVD and related mortality.

Up until the development of the DII, all dietary indices fell into one of three categories: (1) those based on dietary recommendations such as the Healthy Eating Index-2010 (HEI-2010) [51], or the Alternative Healthy Eating Index (AHEI) [52], both based on the US Dietary Guidelines or the Dietary Approaches to Stop Hypertension (DASH) [53], which was promoted by the National Heart, Lung, and Blood Institute; (2) those related to adherence to a particular foodway or cuisine such as the Mediterranean Dietary Index (MDI) [54]; or (3) those derived from a particular study using some kind of regression technique such as principal components analysis or reduced rank regression [55]. These indices have been examined with CVD incidence and mortality as outcome in the past [56]. In a meta-analysis conducted in 2015 that examined results from 15 prospective studies, high-quality diets, which are indicated by having higher HEI, AHEI, and DASH score, showed a significant risk reduction (RR) for CVD incidence or mortality (RR = 0.78, 95% CI 0.75 to 0.81; *p* < 0.00001; *I*^2^ = 45%, 95% CI 13% to 66%) [56]. Higher values for each of these indices represent a healthier diet. Each of them suffers from idiosyncrasies of the approach that include, as a common shortcoming, a narrow range of exposure variability. None was developed specifically to assess the inflammatory potential of the diet. By contrast, the DII was designed to reflect all evidence from a wide variety of human populations as well as from laboratory animal and cell culture experiments. The second advantage of the DII is that it is grounded in research which involved reviewing and scoring 1943 peer reviewed publications that looked at the association between various dietary components and inflammatory markers and is not dependent on a single study or a few studies within the same or similar populations. Results from a cross-sectional study in the USA indicated that the DII score was negatively correlated with the previously mentioned dietary indices (HEI-2010 (*r* = −0.65, *p* < 0.01), AHEI (*r* = −0.55, *p* < 0.01), and the DASH (*r* = −0.52, *p* < 0.01)) [57] and, in large cohort study from Australia, DII was inversely correlated with the Mediterranean Diet Score (MDS) (*r* = −0.45, *p* < 0.01) [58]. Apart from showing strong and consistent association between increasing DII and CVD risk, the DII has also been successfully validated with different markers of inflammation in studies across different populations in different countries [22,25,33,59]. This shows that the DII captures unique properties of diet that are usually not incorporated in a general healthy diet. Presumably, inflammation is the additional aspect of diet that is not captured by other dietary indices and patterns. The 45 dietary components used to derive DII are called food parameters because within this list there are several macro- and micronutrients; food items such as garlic, ginger and onions; and important bioactive polyphenols such as flavonoids. All six groups of flavonoids (isoflavones, flavanol, flavan-3-ol, anthocyanidins, flavones and flavanones) are included in the DII calculation and all of these have a negative literature-derived inflammatory effect score [18].

Multiple theories exist that explain the observed consistent association between DII and CVD risk and related mortality. One of them is the effect of pro-inflammatory diet on increasing levels of cytokines like IL-1 and TNF-α which, in turn, causes attraction and migration of inflammatory cells into vascular tissue [60,61]. These inflammatory markers also increase the expression of cellular adhesion molecules such as selectins and cadherins, which mediate adhesion of white blood cells to the vascular endothelium [62].

A report published from NHANES-III showed that pro-inflammatory diet was associated with an increased risk of CVD mortality in addition to all cause and all-cancer mortality among prediabetic patients [63]. Modified versions of DII have been tested before in relation to CVD risk and mortality. Georgousopoulou et al., in a study from the ATTICA cohort evaluated the association between anti-inflammatory diet and 10-year CVD incidence [64]. In this study, authors calculated a modified version of the DII and have called it Dietary Anti-Inflammation Index (D-AII). The difference between DII and D-AII is that in the D-AII calculation, the *z*-scores are not converted to centered percentiles and instead are directly multiplied by inflammatory effect scores. The scores ranged from 10 to 77. After adjusting for multiple confounding variables, an anti-inflammatory diet, was borderline significantly inversely associated with 10-year CVD incidence (OR 3rd tertile vs. 1st tertile = 0.98, 95% CI: 0.96–1.01) [64]. Finally, in a report from the Spanish cohort of the European Prospective Investigation into Cancer and Nutrition (EPIC) study, the authors again have modified the DII and have called the index Inflammatory Score of Diet (ISD) [65]. The difference between DII and ISD is that instead of standardizing the intake values to means and standard deviations from the global database, authors have standardized the intake values to means and standard deviations of the study population. Subjects classified in the fifth quintile of the ISD (more pro-inflammatory diets) were at higher risk of cardiovascular diseases mortality (RR = 1.89, 95% CI: 1.48–2.40) as compared with those in the first quintile [65].

In addition to its strengths, this meta-analysis also has some limitations. First, in the majority of the studies used for this meta-analysis, DII was derived from dietary assessment tools that were based on self-report using food frequency questionnaires and 24-h recalls, which carries an inherent degree of recall bias and can lead to a potential misclassification of the exposure. Second, the DII score was calculated only once at baseline and human diet might change during study follow-up. However, previous research has shown that adult dietary patterns seem to be relatively stable over time [66]. Third, substantial heterogeneity was observed across studies pooling the CVD risk and mortality. One possible explanation for this could be the differences in the number of food parameters used to calculate the DII score in different studies; another reason could be the different demographic characteristics, and follow-up duration across these studies. Finally, only four studies were eligible for the analysis on CVD risk and six studies for the analysis on CVD mortality.

In conclusion, results from this meta-analysis of a large pool of DII and CVD risk and mortality studies suggests that increasing inflammatory potential of diet as evidenced by higher DII score was independently associated with the increased risk of CVD and related mortality. Hence, educating and encouraging people to consume diets rich in anti-inflammatory food components and low in pro-inflammatory components should play a crucial role in in reducing the risk of developing CVD. Future steps would include exploring how a DII compliant dietary intervention (i.e., aiming to lower overall DII score) would help in preventing newly incident CVD in high-risk populations and in preventing recurrence among those already living with a CVD.

## Figures and Tables

**Table 1:** Main characteristics of the studies included in the meta-analysis.

Author, Year	Study Design	Study Name, Country	Follow-Up (Years)	Cases; Total Population	Cases/Controls or Person Years of Observations or Total Number of Subjects for Lowest Quantile of DII	Cases/Controls or Person Years Observations or Total Number of Subjects for Highest Quantile of DII	Gender	Adjustments
Garcia-Arellano, 2015 [10]	Cohort	PREDIMED, Spain	4.3	277 incident cases; 7216	49/7641 ^a^	79/7960	MF	Age and sex, overweight/obesity, waist-to-height ratio, total energy intake, smoking status, diabetes, hypertension, dyslipidemia, family history of premature cardiovascular disease, physical activity and educational level.
O’Neil, 2015 [38]	Cohort	GOS, Australia	5	76 incident cases; 1363	NA	NA	M	Age, diabetes, systolic and diastolic blood pressure (BP), smoking history, activity level, waist circumference, and total daily energy consumption.
Ramallal, 2015 [39]	Cohort	SUN, Spain	8.9	117 incident cases; 18,794	24/41,240 ^a^	37/42,345	MF	Age, cardiovascular risk factors (hypertension, dyslipidemia, diabetes, smoking status, family history of cardiovascular disease), total energy intake, physical activity, body mass index (BMI), educational level, and other cardiovascular diseases (tachycardia, atrial fibrillation, aortic aneurysm, pulmonary embolism, deep vein thrombosis, peripheral artery disease, heart valve disease, or pacemaker placement), special diet at baseline, snacking, average time sitting, average time spent watching television.
Shivappa, 2015 [29]	Cohort	IWHS, USA	14	6528 CVD deaths; 37,525	1615/195,996 ^a^	1665/192,198	F	Age, BMI, smoking status, pack-years of smoking, HRT use, education, prevalent diabetes, prevalent hypertension, prevalent heart disease, prevalent cancer, total energy intake.
Shivappa, 2015 [43]	Cohort	NHANES III, USA	13.5	1235 CVD deaths; 12,366	368/4183 ^b^	437/4119	MF	Age, sex, race, diabetes status, hypertension, physical activity, BMI, poverty index, and smoking.
Shivappa, 2015 [40]	Cohort	SMC, Sweden	15	2399 CVD deaths; 33,747	445/*n*	560/*n*	F	Age, energy intake, BMI, education, smoking status, physical activity, and alcohol intake.
Neufcourt, 2016 [48]	Cohort	SUVIMAX, France	11.4	292 CVD incidence; 7743	63/22,432 ^a^	89/21,471	MF	Sex, energy intake without alcohol, supplementation group, number of 24-h records, education level, marital status, smoking status, physical activity, BMI.
Vissers, 2016 [49]	Cohort	ALSWH, Australia	11	526 CVD incidence; 6972	71/1626 ^b^	264/5346	F	Age, energy, diabetes, hypertension, smoking status, education, menopausal status and HRT use, physical activity and alcohol consumption.
Wirth, 2016 [50]	Cross-sectional	NHANES, USA	NA	1734 prevalent cases; 15,693	505/3393	373/3531	MF	Family member smoking status, personal smoking status, age, and BMI.
Boden, 2017 [9]	Nested Case-control	NSHDS, Sweden	6.4	1389 acute myocardial infarction cases; 5555 matched controls	210/1056 ^c^	344/1056	MF	Total energy intake, BMI, physical activity, systolic blood pressure, total serum cholesterol, diabetes, smoking, and postsecondary academic education.
Bondonno, 2017 [37]	Cohort	CAIFOS, Australia	15	269 deaths; 1304	55/4368 ^b^	83/4072	F	Age, BMI, energy intake, energy expended in physical activity, socioeconomic status, use of low-dose aspirin, use of antihypertensive medication, use of statins, current or previous smoking, prevalent ASVD (atherosclerotic vascular disease) and treatment.
Shivappa, 2017 [44]	Case-control	NA, Italy	NA	682 cases; 682 controls	154/171 ^c^	225/171	MF	Age, sex, and total energy intake, education, tobacco smoking, BMI, occupational physical activity at age 30–39, coffee consumption, history of hypertension, history of hyperlipidemia, history of diabetes and family history of acute myocardial infarction in first-degree relatives.
Shivappa, 2017 [42]	Cohort	MONICA/KORA, Germany	For CVD mortality: 25.8 and 16.7 years for Survey 1 and Survey 3 For CVD incidence: 21.4 and 13.9 years for S1 and S3	399 CVD related deaths; 1297 men 213 incidence cases; 1297 men	50/324 ^b^ 40/324	74/324 66/324	M	Age, survey, BMI, place of residence, actual hypertension, education level, diabetes, physical activity, energy intake, ratio of total cholesterol and HDL cholesterol, smoking status.
Shivappa, 2017 [41]	Cohort	Whitehall II, UK	22	264 CVD deaths; 7627	84/2456 ^b^	107/2434	MF	Age, sex and ethnicity, occupational grade, living alone, smoking habits, alcohol consumption, physical activity, BMI, antecedent of CVD, use of lipid-lowering drugs, HDL-cholesterol, hypertension, type 2 diabetes and longstanding illness.

^a^ Denominator is person years of observations; ^b^ Denominator is the total number of subject in the DII category; ^c^ Denominator is the total number of controls in the DII category. DII^®^: Dietary Inflammatory Index; PREDIMED: Prevención con Dieta Mediterránea; GOS: Geelong Osteoporosis Study; SUN: Seguimiento Universidad de Navarra; IWHS: Iowa Women’s Health Study; NHANES: National Health and Nutrition Examination Survey; SMC: Swedish Mammography Study; SUVIMAX: Supplémentation en Vitamines et Minéraux AntioXydants; ALSWH: The Australian Longitudinal Study on Women’s Health; NSHDS: Northern Sweden Health and Disease Study; CAIFOS: Calcium Intake Fracture Outcome Study; NA: not applicable; CVD: cardiovascular disease. HRT: hormone replacement Therapy; HDL: high density lipoproteins; M: male, MF: female.

**Table 2:** Analyses of studies exploring the association between DII and CVD, total and individual outcomes. Risk estimates refer to the highest vs. lowest (reference) category of DII.

Subgroup/Additional Analysis	No. of Datasets (Studies)	RR (95% CI)	*I*^2^	*p*
CVD risk and mortality				
Total	12 (12)	1.36 (1.19, 1.57)	65%	<0.001
CVD risk				
Total	6 (6)	1.35 (1.11, 1.63)	36%	0.16
IHD/CHD	3 (3)	1.18 (0.89, 1.58)	37%	0.20
Stroke	3 (3)	1.10 (0.60, 2.00)	65%	0.06
Myocardial infarction	5 (4)	1.43 (1.09, 1.89)	38%	0.17
Angina pectoris	2 (2)	0.79 (0.56, 1.12)	0%	0.73
CVD mortality				
Total	6 (6)	1.37 (1.11, 1.70)	77%	<0.001
CHD mortality	3 (3)	1.37 (0.88, 2.12)	68%	0.05

IHD: Ischemic Heart Disease; CHD: Coronary Heart Disease.

**Table 3:** Subgroup analyses of studies exploring the association between DII and CVD (either risk or mortality). Risk estimates refer to the highest vs. lowest (reference) category of DII.

Subgroup/Additional Analysis	No. of Datasets (Studies)	RR (Relative Risk) (95% CI)	*I*^2^	*p*
Study design				
Cross-sectional	1 (1)	1.30 (1.06, 1.58)	NA	NA
Prospective cohort	11 (11)	1.38 (1.18, 1.62)	68%	<0.001
Sex				
Male	2 (2)	0.95 (0.70, 1.30)	39%	0.20
Female	5 (5)	1.39 (1.05, 1.82)	86%	<0.001
Geographical area				
North America	3 (3)	1.25 (1.03, 1.51)	75%	0.02
Europe	6 (6)	1.37 (1.16, 1.61)	0%	0.51
Australia	3 (3)	1.58 (0.93, 2.67)	81%	0.005
Follow-up duration				
<10 years	3 (3)	1.85 (1.36, 2.51)	0%	0.89
≥10 years	9 (9)	1.30 (1.12, 1.49)	66%	0.003
Adjusted for smoking				
No	0	NA	NA	NA
Yes	12 (12)	1.36 (1.19, 1.57)	65%	<0.001
Adjusted for BMI				
No	2 (2)	1.33 (0.71, 2.51)	67%	0.08
Yes	10 (10)	1.39 (1.19, 1.61)	69%	<0.001
Adjusted for education				
No	5 (5)	1.54 (1.29, 1.83)	41%	0.15
Yes	7 (7)	1.20 (1.04, 1.37)	33%	0.17
Adjusted for physical activity				
No	2 (2)	1.16 (0.98, 1.37)	60%	0.11
Yes	10 (10)	1.44 (1.23, 1.67)	42%	0.08

## Supplementary Materials

The following are available online at http://www.mdpi.com/2072-6643/10/2/200/s1, Figure S1: Funnel plot for (a) CVD (cardiovascular disease) occurrence and mortality, (b) CVD occurrence and c) CVD mortality for the highest versus lowest (reference) category of dietary inflammatory index (DII^®^); Figure S2: Funnel plot for (a) CVD occurrence and mortality, (b) CVD occurrence and (c) CVD mortality for 1-point increase of DII.
