# Supplementary material for: Dietary Inflammatory Index and Cardiovascular Risk and Mortality—A Meta-Analysis
Source: Nutrients. 2018 Feb 12;10(2):200. doi: 10.3390/nu10020200 (PMC5852776; doi:10.3390/nu10020200)

**Supplementary Materials:**

**Figure S1.** Funnel plot for a) CVD occurrence and mortality, b) CVD occurrence and c) CVD mortality for the highest versus lowest (reference) category of DII.


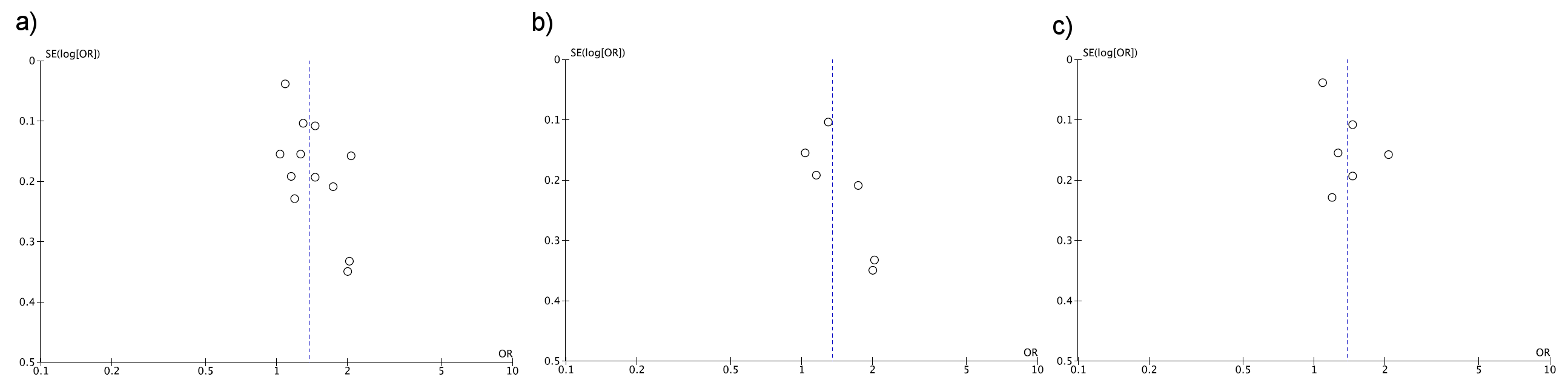


**Figure S2.** Funnel plot for a) CVD occurrence and mortality, b) CVD occurrence and c) CVD mortality for 1-point increase of DII.


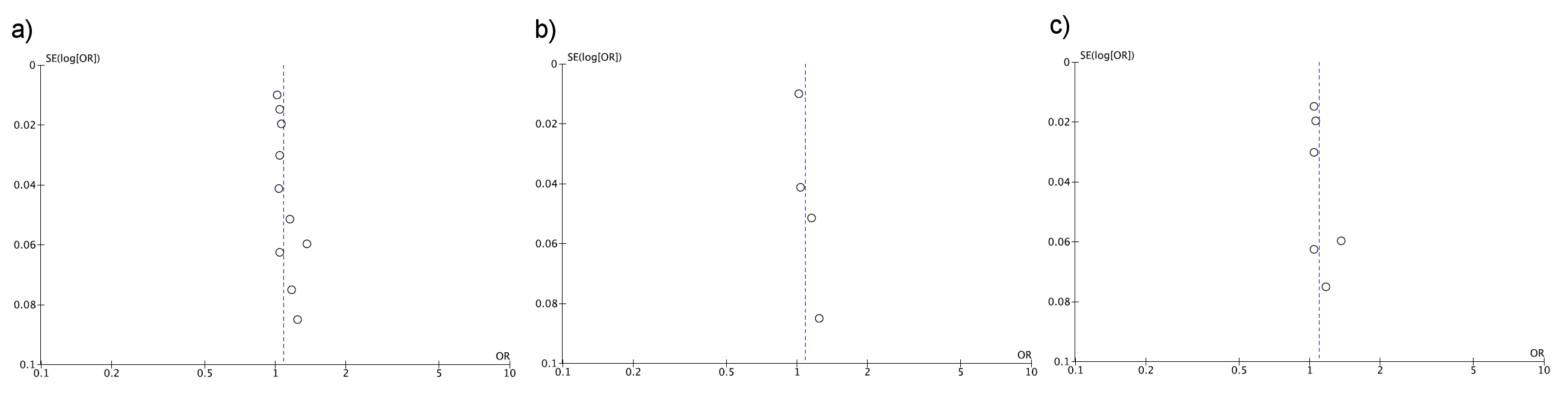

Supplement: Supplementary file 1 [file nutrients-10-00200-s001.docx]
